# Polarized Ukraine 2014: opinion and territorial split demonstrated with the bounded confidence XY model, parametrized by Twitter data

**DOI:** 10.1098/rsos.171935

**Published:** 2018-08-01

**Authors:** Maksym Romenskyy, Viktoria Spaiser, Thomas Ihle, Vladimir Lobaskin

**Affiliations:** 1 Department of Life Sciences, Imperial College London, London SW7 2AZ, UK; 2 Department of Mathematics, Uppsala University, Uppsala 75106, Sweden; 3 School of Politics and International Studies, University of Leeds, Leeds LS2 9JT, UK; 4 Institute of Physics, University of Greifswald, Felix-Hausdorff-Str. 6, Greifswald 17489, Germany; 5 School of Physics, University College Dublin, Belfield, Dublin 4, Ireland

**Keywords:** Ukraine, political polarization, opinion dynamics, bounded confidence XY model, Twitter, natural language processing

## Abstract

Multiple countries have recently experienced extreme political polarization, which, in some cases, led to escalation of hate crime, violence and political instability. Besides the much discussed presidential elections in the USA and France, Britain's Brexit vote and Turkish constitutional referendum showed signs of extreme polarization. Among the countries affected, Ukraine faced some of the gravest consequences. In an attempt to understand the mechanisms of these phenomena, we here combine social media analysis with agent-based modelling of opinion dynamics, targeting Ukraine's crisis of 2014. We use Twitter data to quantify changes in the opinion divide and parametrize an extended bounded confidence XY model, which provides a spatio-temporal description of the polarization dynamics. We demonstrate that the level of emotional intensity is a major driving force for polarization that can lead to a spontaneous onset of collective behaviour at a certain degree of homophily and conformity. We find that the critical level of emotional intensity corresponds to a polarization transition, marked by a sudden increase in the degree of involvement and in the opinion bimodality.

## 1. Introduction

**Figure 1.**
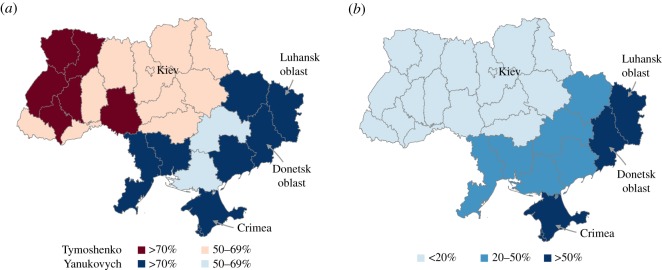
(*a*) Ukraine's political divide in 2010 elections [2]. The majority of the voters in the eastern and southern regions of Ukraine supported a pro-Eastern candidate, Viktor Yanukovych. (*b*) Ukraine's linguistic divide according to national census 2001 [3]. In the three regions most affected by Ukraine crisis in 2014, Luhansk oblast, Donetsk oblast and Crimea, Russian is a native language for more than 50% of the population.

Ukraine represents a bright example of a nearly evenly split society with two opposing camps, where the east/south gravitates towards Russia, while the west/north towards European neighbours [1]. The overall political vector in the country sways between political parties and leaders that on the one hand seek closer ties to the West and in particular Europe and on the other hand to the East and in particular Russia. The Orange Revolution in 2004 brought pro-Western politicians to power; however, in the 2010 elections a pro-Eastern politician, Viktor Yanukovych, was elected president, not least because of major support in the eastern regions of Ukraine (figure 1).

In November 2013, after Yanukovych failed to sign a political association and free trade agreement with the European Union, protests in Ukraine erupted. The initially peaceful rallies became violent in January 2014 after the government passed laws to suppress the protests. In February 2014, the violence escalated, which led to the removal of Yanukovych from office by the parliament. Meanwhile, a separatist and anti-interim-government movement rose with the support of Russia in eastern and southern parts of Ukraine, and ignited a military conflict. Crimea was annexed by the Russian Federation after a referendum that was denounced internationally as illegitimate and illegal. Later in 2014, the crisis resulted in further territorial separation with over 2.6 million internally displaced persons and refugees and a formation of self-proclaimed states in Donetsk & Luhansk [4]. These events escalated the polarization in the country that has grown over the years. As the two political sides became more extreme in their views in the course of the events, a dialogue and therefore a peaceful solution has become increasingly difficult. The extreme opinion divide not only affected society as a whole but also destabilized multiple families and local communities.

In this paper, we present a novel approach, in which we combine rich social media data with the power of methods of statistical physics, to study political opinion polarization mechanisms, seeking to understand what mechanisms turned Ukraine into an irreconcilably polarized state. Though the integration of the two approaches has been increasingly discussed [5,6], there are only few studies so far that have actually attempted to combine these [7–11]. None of them, however, studies polarization and integrates the unstructured data analysis comprehensively with the computational model. In the past studies, there has been a methodological gap between the social media analysis and agent-based modelling, which has limited the relation between the two to rather qualitative statements. We are presenting an approach here that elaborately links the two approaches. Combining them has the advantage to parametrize the computational model and validate it by empirical evidence and on the other hand to make use of the rich social media data in a theory-guided way beyond mere descriptives. This holds the potential to gain new insights into the underlying social mechanisms of polarization. In the following, we will describe a new computational model of polarization, a two-dimensional lattice agent-based model, that is informed by the theoretical work on polarization discussed below and that brings in the spatial dimension and emphasizes the role of regional differences. We then look at the empirical polarization dynamics in the Ukrainian Twittersphere, before parametrizing the model with the analysed Twitter data, hereby validating the model. The combination of the two approaches reveals the important role that emotional intensity levels play in polarization thus far not sufficiently accounted for by classical theoretical or empirical studies of polarization. The few studies that investigate the role of emotions in polarization have usually focused on very specific emotions, e.g. ‘self-conscious’ emotions like pride and embarrassment/shame, and showed that these emotions can reinforce conformity and polarization [12]. In this paper, we do not focus on specific emotions, but rather examine the role of emotional intensity levels and show how these emotional intensity levels can be a decisive driver in polarization.

## 2. Opinion polarization

Opinion polarization has been intensely studied over the last few decades, initiated by the observation that groups tend to adopt positions that are more extreme than the initial individual positions of its members [13,14]. One explanation of this phenomenon is based on the Social Comparison Theory, which suggests that people want to be perceived in a more favourable way than what we perceive to be the average tendency. Through observations they determine what the average tendency is and then they express a slightly more extreme opinion than the perceived average opinion [13,15,16]. There is clear evidence for this assumption from numerous experimental studies [13,17,18]. Moreover, due to the homophily phenomenon [19], which states that people are more likely to interact with those who are similar to them with respect to socio-economic background [20] as well as attitudes [21], people are more likely to socially compare themselves to similar peers. Group polarization phenomena are also explained drawing on the Persuasive Argument Theory, which states that people are more likely to change their opinion when presented with persuasive arguments. Group polarization can occur when the group discourse is manipulated through biased information or misinformation, exposing a group to false persuasive arguments [13,16,22]. The theory is strongly supported by empirical evidence from numerous experimental studies [13,23,24]. The two mechanisms, social comparison and persuasive argument, usually co-occur. For instance, persuasive arguments in an environment of biased information can further push an individual to adopt a more extreme attitude than expressed in the group they compare themselves to. Another mechanism with respect to polarization dynamics is the Biased Assimilation [25] in opinion formation processes, which maintains that people are likely to keep their original position and draw support for it if confronted with mixed or inconclusive arguments. This mechanism, which again is empirically well established [26–28], shows that people are only to a limited extent open to change their opinions and this can contribute to polarization dynamics. In fact, Dandekar *et al.* [21] show that the two earlier described mechanisms, and in particular the social comparison mechanism, is not sufficient to produce polarization; the biased assimilation mechanism has to be added.

Polarization processes have been empirically investigated through experimental studies as mentioned previously and to a lesser extent through survey-based studies [29,30]. Furthermore, applied statistical physics and agent-based modelling approaches [21,31–33] have been used extensively to study polarization mechanisms through computer simulations. Prominent are, for instance, bounded confidence models of opinion dynamics, stochastic models for the evolution of continuous-valued opinions within a finite group of individuals that explore conditions for consensus and opinion fragmentation, introduced by Deffuant *et al.* [34] and further elaborated by numerous studies [35–37]. We will also draw inspiration from these models. The challenges of using statistical physics models for modelling social phenomena are known. The tractable models are usually oversimplified and too general and thus lack flexibility required to reflect the features of a particular social phenomenon. Moreover, while computational studies are rigorous in investigating the specific mechanisms and dynamics of polarization, they often lack empirical foundation and thus it remains often unclear to what extent these often abstract models accurately represent phenomena we see in the real world. More recently, social media (e.g. Twitter and Facebook) data have been increasingly used to study opinion polarization [38–41]. Gruzd & Tsyganova [42], for instance, use Ukrainian Vkontakte data to demonstrate the split between the two political camps in Ukraine (pro-East versus pro-West) during the Maidan protests. Twitter has also played a quite important role as a contested public debate arena throughout the crisis in Ukraine [43,44]. Studies with social media data have revealed the strong effect of homophily in online social networks, which may lead to phenomena like the echo chamber [41,45,46], where opinions are amplified through communication and repetition inside an ‘enclosed’ social system. Echo chambers can prevent people from noticing contrary persuasive arguments and they skew the perceived average that people take into consideration in social comparison processes. Though social media data are potentially rich, i.e. fine-grained, time-resolved, relational, geo-coded, etc., without an explicit theoretical underpinning, the studies of polarization on social media remain usually rather descriptive. By combining rich social media data with the power of methods of statistical physics, a better understanding of specific political opinion polarization mechanisms is sought in this paper.

## 3. Methods

### 3.1. Political opinion mining of Twitter data

**Figure 2.**
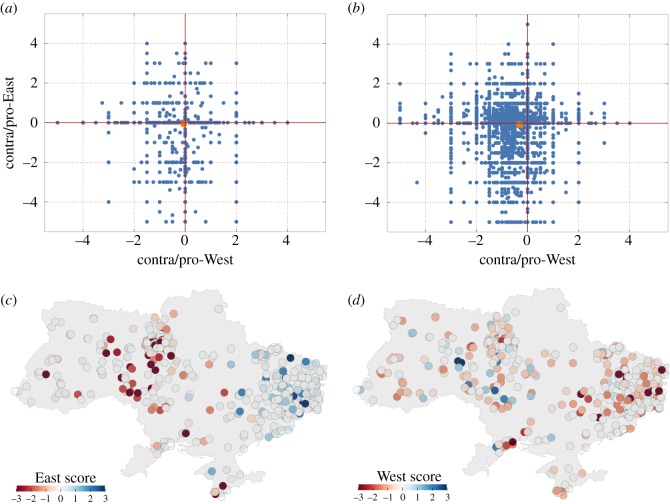
(*a*) Pro-against-European (West)/pro-against-Russian (East) opinion space plot for the first week of October 2013. (*b*) Pro-against-European (West)/pro-against-Russian (East) opinion space plot for the second week of August 2014. The points in plots *c* and *d* are Twitter users based on their two scores, the orange dot is the average. (*c*) Twitter users (dots) coloured according to their ‘East’ affiliation score and plotted over the Ukraine map using Twitter geo, place or location information. (*d*) Twitter users (dots) coloured according to their ‘West’ affiliation score and plotted over the Ukraine map using Twitter geo, place or location information.

We used the archived Twitter API Streaming data from October 2013 to September 2014 provided by the Archive Team [47]. The archived data are the freely available Twitter Streaming API Spritzer Sample, which collects 1% of all public tweets in real time. The Twitter Streaming API Spritzer Sample allows for unfiltered data analysis and hence for capturing the full discourse picture, compared with a more narrow, focused (e.g. based on certain hashtags and user networks) approach facilitated by data using the Twitter REST API [48] that would disregard discourse contributions beyond the specified search queries. The data for January 2014 were missing and we did not find any other archive providing these data. The tweet data are stored in JSON format. The data were processed and analysed in Python. Specifically, the data were filtered for Russian/Ukrainian language (excluding Twitter users who specified being from Russia), cleared of SPAM, and then filtered for political content with an extensive set of keywords (see the electronic supplementary material for details). We used the tweets to determine the political affiliation of each Twitter user using a set of keywords indicating neutral, pro- or anti-West attitudes and a set of keywords indicating neutral, pro- or anti-Russian attitudes (see the electronic supplementary material for details). Moreover, we conducted a sentiment analysis of the tweets, in order to determine the political affiliation for previously seemingly neutral Twitter users. For this purpose, we used the SentiStrength system for automatic sentiment analysis, built a Ukrainian sentiment words dictionary (see the electronic supplementary material) and extended the SentiStrength Russian sentiment words dictionary to make it equivalent to the Ukrainian sentiment words dictionary (see the electronic supplementary material for details). Our classification algorithm scanned all tweets for each user and checked whether the tweet contained any of the keywords and any of the sentiment words specified and calculated overall scores for political ‘West’ and ‘East’ affiliation as well as sentiment scores (see the electronic supplementary material for details). For the geo-plots in figure 2*c*,*d*, we focused on the data of the last week in September 2014. We scanned the Twitter data of each user for two possible geographical information, the value of their ‘location’ tag and/or geo/place ‘coordinates’ tag which is a latitude and a longitude coordinate value. The vast majority of users have the default option ‘geo_enabled:false’, thus do not provide precise geographical information attached to the tweet, a few more provide profile ‘location’ information, but overall, geographical data are often not specified and thus missing. We therefore ended up with only 493 Twitter users for whom we had geographical information. The ‘West’ and ‘East’ political affiliation scores of these 493 Twitter users were plotted in figure 2*c*,*d* (see the electronic supplementary material for details).

### 3.2. Bounded confidence XY model of opinion polarization

We model opinion dynamics in an assembly of interactive agents placed on a two-dimensional lattice of finite size, which emulates a regional opinion distribution and localization. Each opinion is represented by a vector that can freely rotate in plane, similar to that in the XY model of a magnet, and is characterized by the length *p* (emotional intensity, i.e. fervent strength of an opinion) and polar angle *θ* (orientation). This allows us to model a continuous opinion spectrum with respect to the specified direction by a cosine of the angle between the vectors, which can vary in the range −1 (agent opposes the opinion) and 1 (agent fully supports the opinion). Each agent can interact with a fixed number of nearest neighbours. The social interactions are introduced via local mean field, similar to the Vicsek model [49]: the new orientation of each vector is calculated as a mean of the average direction of the neighbours' and agent's own orientation:

3.1 $$\theta_{i}(t+\Delta t)=\tan^{-1}\left[\frac{1}{N}\frac{\sum_{j,|j-i|\leq r}p_{j}\sin(\theta_{j}(t))}{\sum_{j,|j-i|\leq r}p_{j}\cos(\theta_{j}(t))}\right]+\xi (\eta ,t),$$

where *N* is the number of interacting agents, *ξ* is the angular noise variable uniformly distributed in the interval [ − *η*/2, *η*/2] and *η* is the noise strength. The noise is added to model the level of conformity of the individual: zero noise, *η* = 0 corresponds to full conformity, *η* = 2*π* allows an individual to deviate from the group opinion by an unlimited value. A contribution of each interacting agent is weighed by the emotional intensity of its opinion *p*. The interaction has a limited range *r*, which sets the number of nearest neighbours considered. In two dimensions, interaction range *r* = 2 corresponds to 24 interaction peers, *r* = 3 gives 48 peers, etc. (see the electronic supplementary material for details). In addition to this, the interaction is selective so that the vectors align only with those neighbours whose orientation (opinion) deviates by an angle less than some fixed value *α* from their own opinion vector. This rule is inspired by the bounded confidence model, or Deffuant model [34], and allows one to imitate systems with different levels of opinion tolerance [50]. Scaled value *α*/*π* denotes a fraction of the opinion spectrum that is taken into account by each individual, e.g. *α*/*π* = 0.1 corresponds to 10% closest opinions taken into account. Note that our model does not explicitly include any intrinsic preference to agent's own opinion (i.e. orientation of the opinion vector *i* in the previous time step) because using relatively small values of *η* and *α* already allows to achieve a socially realistic behaviour.

We model a finite system with the boundary conditions set by two fixed rows of agents on each side. The boundary vectors are fixed according to the following scheme: the left and upper rows are oriented to the left (*θ* = *π*), imitating a bias towards ‘West’ and the right and bottom rows are fixed to point in the opposite direction (*θ* = 0), thus mimicking a bias towards ‘East’. When other agents interact with the boundary vectors, this introduces a spatially dependent local bias that can account for geographical inhomogeneity in opinions, thus imitating cultural or ethnic differences, information bias, etc. (see the electronic supplementary material for details; see also the interactive model [51]).

These model settings reflect the theoretical assumptions about polarization described above. The orientation represents political attitudes, and the local mean field calculation of new orientations for each agent represents the social comparison theory assumptions. The homophily effect is included through the bounded confidence feature, where agents align only with those neighbours whose political orientation is similar to their own. Furthermore, the biased assimilation mechanism is reflected in the noise variable that determines the agents' willingness to change opinion (conformity level). Finally, the persuasive argument theory assumption, in particular, with respect to the contribution of biased information to polarization, is simulated via the boundary conditions. We have added a further parameter to our model, the emotional intensity, i.e. vector length, that represents the level of emotional strength and vehemence of a political opinion. This parameter reflects results we have obtained from Twitter data sentiment analysis discussed in the next sections. It also builds on recent computational models and big data analysis of opinion dynamics [52,53], which showcase the importance of emotions, and in particular of negative emotions, for people's engagement in political debates and for opinion formations and changes.

We modelled evolution of the opinion spectrum in the described system starting from randomized initial distributions of agents' emotional intensity and orientations based on general uniform distributions. The emotional intensity levels for each agent were kept fixed in each simulation, while the orientations evolved due to noise and interactions. For each opinion spectrum extracted from Twitter data, we performed a simulation until a steady state was reached. After that, the following statistics were collected: steady-state distribution of the opinion along the ‘East–West’ scale, as defined by the boundary conditions, mean order parameter and bimodality index.

We should note here that in this set-up the week-by-week series of calculated properties reflect neither the real time nor the actual system's dynamics as the history of the individuals as well as previous steady states are ignored. To follow the variation of collective properties, ideally one should look at the evolution of each user's opinion and derive the group behaviour from the corresponding statistics. For this purpose, one would need to either parametrize the individual opinions from the empirical data directly or solve the inverse problem and introduce the variation of opinions based on the instantaneous statistical averages. As we could reach only a random sample of the tweets, it was not possible to follow the former route and track individual users. The empirical data we have are discontinuous. Moreover, we have no appropriate model for individual psychology. Therefore, we decided not to introduce an artificial evolution of the opinions. As the individual history is lost, we can only follow the variation of the averages corresponding to the snapshot of Twitter data.

### 3.3. Model parametrization with Twitter data

To parametrize the bounded confidence XY model, we used weekly distributions of the overall users' emotional intensity scores. For each week, the overall emotional intensity of each user was defined from the mean of the average sentiment scores on a continuous scale from 0 to 5, with 0 corresponding to a neutral average user's opinion and 5 reflecting an extremely emotional average opinion. This measure did not contain any information about political affiliation, so that each non-zero value could correspond to either pro-West or pro-East user's political attitude. The data were sampled with a bin size of 1 for all non-zero opinions and a resulting discrete distribution was normalized by the total number of users per week giving probabilities for each discrete value of overall emotional intensity (0 to 5). This distribution was then applied to the simulated system to define the length *p* of each opinion vector. In each simulation, values of *p* were assigned to agents at random, according to the obtained discrete weekly distributions of overall emotional intensity, and kept constant throughout the simulation. The simulated system consisted of 14 641 agents placed in nodes of 120 × 120 lattice. We performed at least 10^6^ update cycles to determine the structure of the steady state. Each statistic was averaged over at least 5 independent runs. We computed the bimodality coefficient as *β* = (*γ*^2^ + 1)/*k*, where *γ* is the skewness and *k* is the kurtosis of weekly distributions of average opinion scores in Twitter data or weekly distributions of cosines of orientations *θ* of opinion vectors in simulations.

## 4. Results

### 4.1. Opinion polarization in the Ukrainian Twittersphere

To validate the model assumptions, we used Twitter Streaming data from October 2013 to September 2014 provided by the Archive Team. We use Twitter data because it provides fine-grained, time-series and rich data on recent political opinion dynamics in Ukraine, otherwise not available. To determine the political affiliation of the Twitter users in our data, we used a political affiliation classification procedure based on keyword and sentiment analysis suggested and tested by Spaiser *et al.* [54] (see the electronic supplementary material for details). As a result, every Twitter user was assigned two scores, a ‘West’ score and an ‘East’ score, representing their political position in a pro-against-European (West)/ pro-against-Russian (East) opinion space (figure 2*a*,*b*) and an emotional intensity score, combining their sentiment analysis scores.

**Figure 3.**
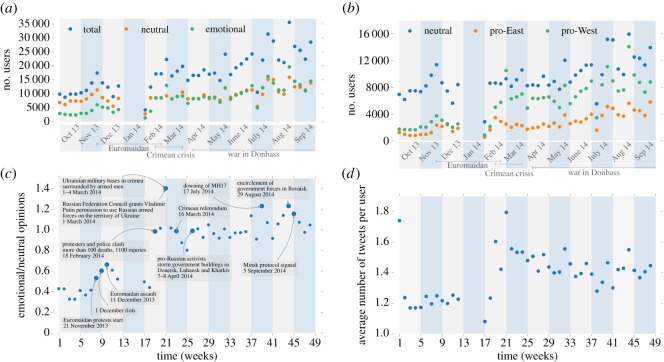
(*a*) Time series plot of number of tweets, in total, neutral and emotional. (*b*) Time series plot of emotional/neutral tweets ratio with key political events tags. (*c*) Time series plot of number of average (weekly) users' opinions, in total, pro-West and pro-East. (*d*) Average weekly number of tweets per user. No data were available for January 2014 (see Materials and methods and the electronic supplementary material) therefore the data gaps in plots (*a*–*d*) between the weeks 13 and 17.

Our Twitter analyses show that political polarization did indeed take place in the Ukrainian Twittersphere between October 2013 and September 2014. Figure 3*a* shows a discontinuous increase in emotional users in February 2014, while the total number of users and the number of neutral ones increased steadily. Figure 3*b* depicts a ratio of emotional/neutral opinions, where a jump from values of about 1.0 to about 1.4 is visible around February. We added to this figure the key political incidents in Ukraine during this year, so the discontinuous changes in the data can be related to actual political events. This shows that the biggest discontinuous change in February took place around the time when the Maidan protests escalated and 100 people died on a single day. These results inspired the inclusion of emotional intensity levels as a parameter in the computational model. In addition to an abrupt increase in the total number of users (figure 3*a*,*c*), users' involvement in the topic also changed discontinuously (figure 3*d*). The dynamics of the average number of tweets per user completely resembles that of the emotional level of tweets (figure 2*b*), with a characteristic jump around week 19 (February 2014).

Figure 2*a*,*b* moreover shows the polarization in a continuous two-dimensional space defined by pro-against-European (West)/pro-against-Russian (East) scores. This figure shows that the polarization resulted in the appearance of two main opinion clusters, those who are pro-East and against-the-West on the one hand and those who are against East but who are also quite critical of the West on the other hand (see the electronic supplementary material for details). This, in fact, reflects most current surveys that show the disappointment of many Western-oriented Ukrainians with the pro-West Poroshenko government [55].

Our Twitter data analysis confirms that political affiliation follows the expected geographical pattern. People who are pro-East and against-West are more likely to be located in the eastern and southern parts of Ukraine, while Ukrainians with rather an against-East and (critical) pro-West attitude are to be found in the western and northern territories (figure 2*c*,*d*). This analysis is a validation of our classification procedure, as the geographical distribution of the Twitter users with their respective political affiliation scores matches the actual geographical political camp distributions in Ukraine (figure 1).

### 4.2. Validating the bounded confidence XY model of opinion polarization with Twitter data

**Figure 4.**
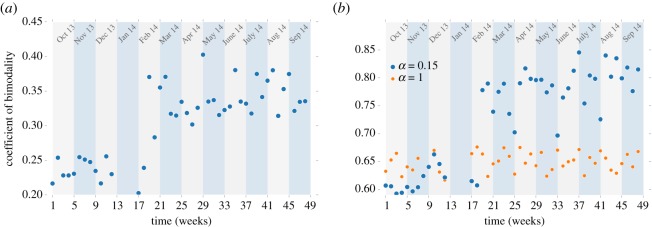
(*a*) Time series plot of coefficient of bimodality of Twitter users' opinion distribution. (*b*) Time series plot of coefficient of bimodality of vector orientations in simulations (*η* = 0.12).

To quantify the opinion divide, we present here the bimodality index for the opinion spectrum (see Materials and methods for the definition), as we found it to be most sensitive to the changes of the spectrum, therefore, the best integral characteristic of the observed behaviour. The changes in the bimodality coefficient over 12 months from October 2013 are shown in figure 4*a*,*b*. In the Twitter data, the bimodality keeps as low as 0.2–0.26 from the start of observation until week 18 (February 2014), but then demonstrates sudden increase to 0.35–0.40 within the next two or three weeks, after which it stays high, and the original value is never restored. This sudden increase of the bimodality reflects a formation of two distinct political camps and clearly shows that the opinion divide has suffered a significant deepening in this short period. Moreover, we see that the deepening was non-recoverable in the short term. The computational model, parametrized with the Twitter emotional intensity spectrum for each respective week, captures this behaviour well and shows a similar qualitative trend. In a simulation with *α* = 0.15*π*, the bimodality coefficient jumps from *ca* 0.60–0.65 to 0.8–0.85 during the same period. The quantitative difference in the values is mostly due to the use of cosine function. We should note that, although the model is parametrized by the Twitter data, it is not bound to reproduce the distribution, as the vectors are allowed to change the orientation, and this behaviour follows only from the specific anisotropic interactions between the agents. We repeated the simulations with a different interaction parameter *α* = 1.0, corresponding to agents without any resistance to opinion change and found no jump in the bimodality. Therefore, the opinion divide is conditioned by both the restriction of confidence and by conformity (noise) levels, thus confirming the homophily and biased assimilation assumptions. Moreover, the drastic change in bimodality corresponds to the sudden increase of the emotional intensity, which we noted in the data in figure 3*b*.

### 4.3. Onset of opinion clustering and formation of territorial domains

**Figure 5.**
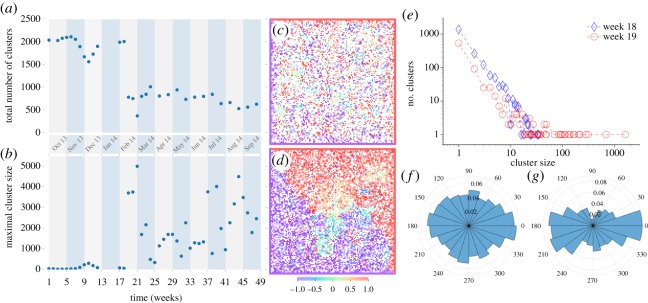
(*a*) Time series plot of average number of clusters in simulations. (*b*) Time series plot of maximal cluster size in simulations. (*c*) Simulation snapshot for week 18, February 2014. (*d*) Simulation snapshot for week 19, February 2014. Each square in plots *c* and *d* represents an individual agent; size of a square is proportional to emotional intensity; colour of each square denotes cosine of an orientation angle for each spin, with −1 and 1 corresponding to orientation towards ‘West’ and ‘East’, respectively. (*e*) Log–log plot of distribution of cluster sizes in simulations for week 18, February 2014 (blue) and week 19, February 2014 (red). (*f*) Simulation cluster orientation diagram for week 18, February 2014. (*g*) Simulation cluster orientation diagram for week 19, February 2014. Orientation of clusters in plots *f* and *g* is shown in degrees; 180 and 0 degrees denote orientation towards ‘West’ and ‘East’, respectively; length of each bin reflects cluster probability. Simulation parameters in plots (*a*–*g*) are *α* = 0.15 and *η* = 0.12.

The increase in emotional intensity leads to important consequences in the spatial dimension. In the weeks before and early at the outbreak of the crisis, the simulations display large diversity of the opinions (figure 5*c*) characterized by the large number (figure 5*a*) of small (figure 5*b*) opinion domains (clusters). This picture, however, changes further into the crisis, around week 19, when a smaller number of larger opinion domains is formed. While clusters rarely exceed 100 agents before the critical weeks 17–18, starting from week 19 we observe clusters of up to 5000 agents and the numbers rarely drop below 1000. That the behaviour becomes expressly collective is consistent with the rise of emotional intensity and of the fraction of involved agents (those with non-zero valence), which increases the number of interactions within each individual's circle and thus the local aligning field. This is further confirmed by figure 5*f* ,*g* showing the polar opinion histogram for the two critical time points, weeks 18 and 19, obtained from simulation analyses. The distributions are visibly gravitating towards 0 or 180 degrees (‘East’ and ‘West’) in both sets. The fraction of neutral opinions drops from week 18 to week 19 and the distribution of opinions in each subgroup becomes very narrow.

Simulation analysis, moreover, shows that the opinion divide induces also territorial splitting. Figure 5*c*,*d* shows the in-plane opinion distribution. We plotted the opinions as predicted by the model just before the jump in the bimodality (week 18, figure 5*c*) and immediately after that (week 19, figure 5*d*). The change in the distribution between these points is dramatic: while the predicted data for week 18 show a merely uniform distribution of both ‘East’ and ‘West’ orientations, the picture for week 19 features two distinct clusters with predominant ‘West’ orientation in the lower left corner and domination of the ‘East’ orientation in the top right corner. These orientations correspond to vector directions in the preset boundary conditions. Each domain contains practically no opposing opinion, as they are squeezed out to the periphery and then to the opposing domain as the steady state develops. We can see small islands of mixed/neutral opinion in the middle of the simulation domain. The prediction of the territorial divide matches also well the geo-location data shown in figure 2*c*,*d*. We should stress, however, that the biased boundary conditions alone are not sufficient to produce any large domains even in the system with limited confidence (figure 5*c*, see the electronic supplementary material for details), although they definitely facilitate this collective behaviour.

The simulation allows us to analyse the steady states of the system and provides insights into the mechanisms of sudden onset of polarization, clustering and territorial splitting (see the electronic supplementary material for details). We, in particular, examined varying levels of noise, restriction angles and vector lengths. High noise (low conformity) corresponds to a globally disordered behaviour (without any prominent consensus or polarization) and the range of higher *α* allows only states with polar order (global consensus). Smaller restriction angles, *α* < 0.4*π*, on the other hand produce regions of prevalence of the bipolar states, thus structure the system in a polar or bipolar way. A combination of small restriction angle (strongly bounded confidence) and high noise (low conformity) does not produce any global order and is rather unrealistic because at these conditions the system represents a set of selective but randomly vacillating agents. Polarized states are generally only possible at low noise (high conformity) and small restriction angles (strongly bounded confidence). The changing level of emotional intensity pushes the boundary between the polarized and non-polarized societies moreover outwards, thus extending the range of the polarized states, and brings the originally weakly polarized society to a highly polarized one. This splitting resembles in appearance a phase separation in dissimilar liquids (e.g. oil in water). The important difference of our system from the liquid-state systems is that the agents are not dissimilar from the beginning, but the dissimilarity and effective repulsion between the opposite arises from strong social interactions that dictate cohesion between the like opinions. Another important observation here is that the transition is driven primarily by the increase of emotional level, while the other parameters (conformity, confidence) stay constant. We should stress that the source of and the original direction of the emotional agents were not crucial. The key properties of the model that determine the nature of the steady states (non-polar, polar or bipolar) are the high conformity and strongly bounded confidence.

## 5. Conclusion

We have analysed the opinion dynamics over a recent period of political unrest in Ukraine. Based on the Twitter data, we registered an onset of emotional intensity of tweets that corresponded to rising levels of involvement of the population in the political feud, fuelled by the action of the government, collisions of the opposing groups and foreign military activities. The escalating opinion divide around the time became apparent among others in the jump of the opinion bimodality index. We proposed an agent-based lattice model to study political polarization as a collective behaviour including the spatial dimension of polarization. By parametrizing the model with Twitter data at distinct time points, we predicted the onset of collective behaviour and territorial splitting of the opinion. We demonstrated that the tendency of territorial splitting is conditioned by the high conformity and homophily in the society and is driven by the growth in emotional intensity. Our analyses demonstrate clearly the importance of emotional intensity for polarization, a factor that has been largely ignored thus far in classic theoretical and empirical literature on polarization with a few noteworthy exceptions as discussed earlier. Specifically, while our analyses confirm the importance of social comparison, homophily, persuasive argument and biased assimilation mechanisms and their specific interactions for polarization, they also show that these mechanisms are not sufficient to ignite the extreme societal polarization we can observe, for instance, in Ukraine. The emotional intensity is a key reinforcement mechanism that has to be added.

Our analysis seems also to suggest a link between polarization and separatist trends. The polarization dynamics that we establish for February 2014 in the data and simulation increases in fact further around April and May 2014, when the self-declared Donetsk and Luhansk People's Republics were formed in the southeast of Ukraine. This seems to suggest that the opinion split may facilitate the separatist trends on its own. The observed phenomenon is not unique to Ukraine, and similar processes of deepening polarization leading to separatism are well known elsewhere (e.g. Northern Ireland). In most cases, however, the separatism is related to more obvious ethnic, racial or religious differences between the communities, while in Ukraine the division is more subtle and roots in small cultural differences, which were artificially enhanced by external factors. And our results show how dangerous targeted agitation can be when it is backed by modern information warfare techniques [56,57] as it boils emotions making polarized world views increasingly irreconcilable.

## Supplementary Material

Supplementary Material for: Polarized Ukraine 2014: Opinion and Territorial Split Demonstrated with the Bounded Confidence XY Model, Parameterized by Twitter Data

## Supplementary Material

Ukrainian Sentiment Dictionary

## Data accessibility

The datasets supporting the conclusions of this article are provided by the Archive Team [47].
