# Supplementary material for: Polarized Ukraine 2014: opinion and territorial split demonstrated with the bounded confidence XY model, parametrized by Twitter data
Source: R Soc Open Sci. 2018 Aug 1;5(8):171935. doi: 10.1098/rsos.171935 (PMC6124111; doi:10.1098/rsos.171935)
Supplement: Supplementary Material for: Polarized Ukraine 2014: Opinion and Territorial Split Demonstrated with the Bounded Confidence XY Model, Parameterized by Twitter Data [file rsos171935supp1.pdf]

Supplementary Material for: Polarized Ukraine 2014: Opinion and Territorial Split Demonstrated with the Bounded Confidence XY Model, Parameterized by Twitter Data

Maksym Romenskyy, Viktoria Spaiser, Thomas Ihle, and Vladimir Lobaskin
(Dated: June 26, 2018)

Supplementary Methods

Further Model Specifications

Supplementary Figure 1 illustrates interactions in the Bounded Confidence XY model. The red opinion vector interacts with its 24 neighbors located within two nearest lattice nodes in any direction $r = 2$. In this illustration, each of the 24 neighbors has orientation relative to the focal (red) vector less than α and therefore all 24 agents contribute to future orientation of the focal vector. For simplicity, all vectors in Supplementary Fig. 1 are shown to have same length. In all simulations, the initial orientations of all agents except the ones at the system's boundary were drawn randomly from the uniform distribution in the interval $[-\pi, \pi]$.

To quantify formation of territorially isolated domains in our model, we performed a simple cluster analysis. We define a cluster based on two criteria: distance and relative orientation between the agents. Therefore, a cluster is a set of connected agents, each of which is within the cutoff distance (defined by r) from one or more other opinion vectors from the same cluster and a relative orientation between any two neighbors in the cluster is less or equal to restriction angle α. Conversely, two agents will not belong to the same cluster, if there is no continuous path on the neighbor network leading from the first agent to the second or if this path is broken because the angle between two neighbors is larger than α and hence the opinion vectors do not interact. For each update step we calculated size of each cluster, maximal cluster size and total number of clusters.

We characterised orientational ordering in our model using two order parameters. Polar order parameter was used to quantify the average degree of opinion agreement between the agents

$$\varphi = \frac{1}{N} \left| \sum_{j=1}^{N} \exp(\imath\theta_j) \right|, \quad (1)$$

where $\imath$ is the imaginary unit and θ_j is the direction of each vector j. This order parameter turns zero in the isotropic phase, when opinions of all agents are very different from each other, and assumes finite positive values in the ordered phase, reaching unity when global consensus settles in.

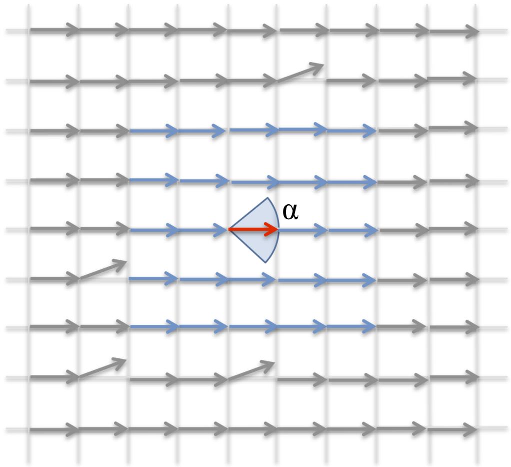

Supplementary Figure 1: The interaction parameters in the Bounded Confidence XY model with $r = 2$. The focal opinion vector (red arrow) interacts only with those neighbors whose relative orientation is less or equal to α and who are located within the two nearest rows/columns (blue arrows).

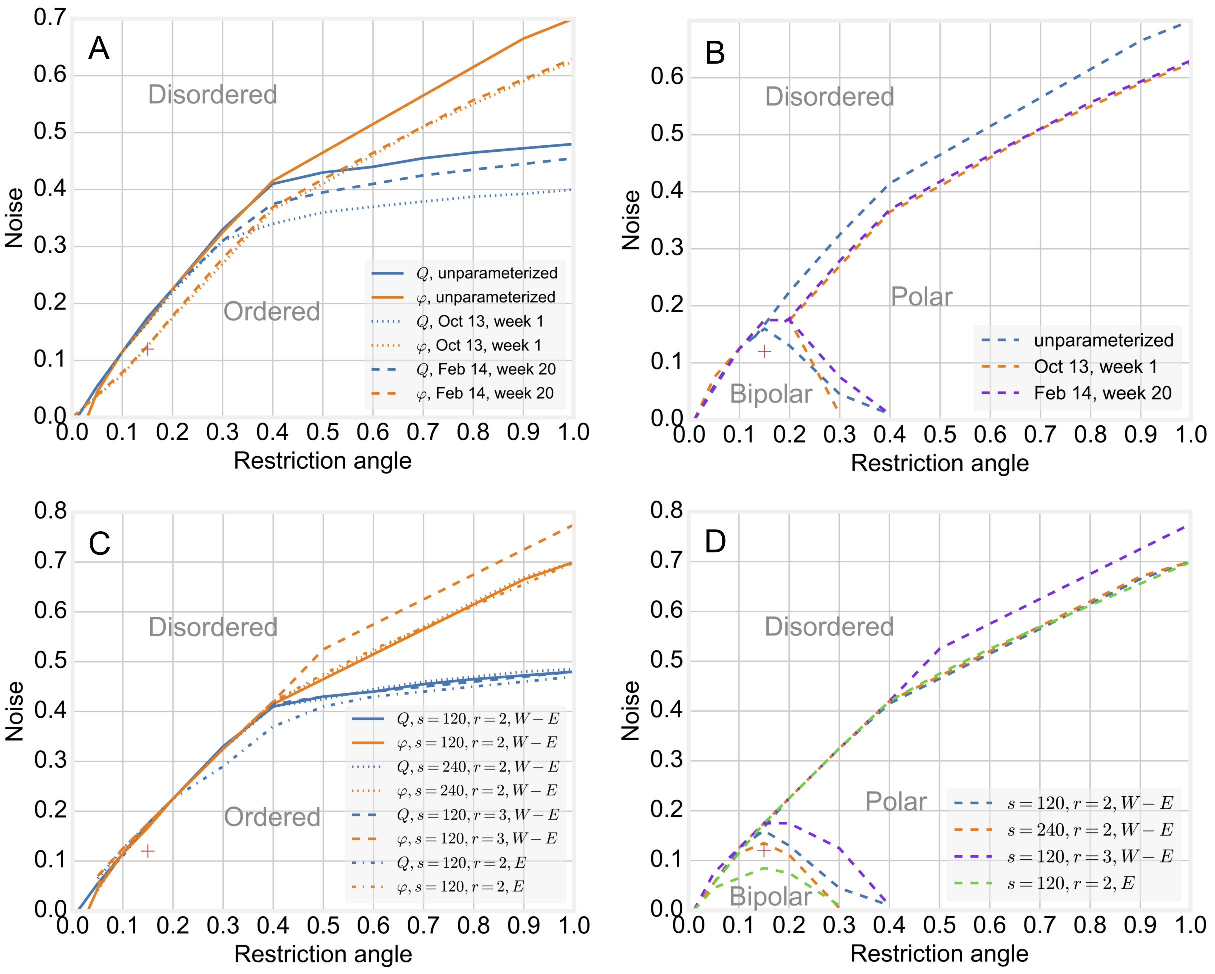

Supplementary Figure 2: Phase behavior of the Bounded Confidence XY model. (A) Phase diagrams based on polar φ and bipolar Q order parameters for the model system without and with parameterization by Twitter data. (B) Combined phase diagrams for the model system without and with parameterization by Twitter data showing regions of dominance of polar and bipolar order. (C) Phase diagrams based on polar φ and bipolar Q order parameters for non-parameterized model system at different simulation parameters (see legend). (D) Combined phase diagrams showing regions of dominance of polar and bipolar order for unparameterized model system at different simulation parameters (see legend). The red plus marker denotes simulation parameters α and η for the parameterized version of the model used throughout the paper. In plots C and D, s denotes size of a side of a square lattice; E stands for East and means that all boundary spins are fixed to the right ($\theta = 0$), $W - E$ stands for West – East and means that the boundary spins on the left and on top are fixed to the left ($\theta = \pi$) and the boundary spins on the right and on the bottom are fixed to the right ($\theta = 0$); r denotes the interaction range.

To characterise opinion polarization in our model we use the following bipolar order parameter

$$Q = \left| \frac{1}{N} \sum_{j=1}^{N} \exp(\imath 2\theta_j) \right| . \tag{2}$$

When the two vectors are oriented perfectly collinearly, $Q = 1$. Note that a perfectly polarly ordered phase is characterized by $\varphi = Q = 1$, as the polar ordering implies the bipolar ordering. A bipolarly ordered phase requires only $Q = 1$ while the polar order parameter can take any value $\varphi < 1$. Therefore, requirements for the polar order are more restrictive.

For each weekly distribution of opinions, both for the Twitter data and in simulations, we computed bimodality coefficient (see main text, Materials and Methods) by first calculating skewness and kurtosis of the distribution.

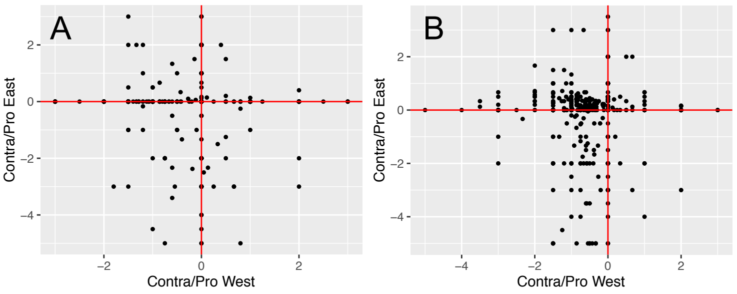

Supplementary Figure 3: (A) Panel Twitter users (black dots) in the pro-against-European (West)/pro-against-Russian (East) opinion space, data from first week in October 2013. (B) Panel Twitter users (black dots) in the pro-against-European (West)/pro-against-Russian (East) opinion space, data from first week in September 2014.

Skewness is defined as the third standardised moment around the mean

$$\gamma = \frac{\mu_3}{\mu_2^{3/2}}, \tag{3}$$

where μ_2 and μ_3 are the second and the third cumulants, respectively. Kurtosis is computed as the fourth central moment

$$k = \frac{\mu_4}{\mu_2^2}, \tag{4}$$

where μ_2 and μ_4 are the second and the fourth cumulants, respectively.

The model phase diagrams, shown in Supplementary Figs. 2 A-D, demonstrate three different steady states of the system. The high noise (low conformity) generally corresponds to a globally disordered behavior (without any prominent consensus or polarization). The region of disordered behavior shrinks as the restriction angle α is increased (Supplementary Fig. 2 A). The lesser restriction leads to more interactions between the nearest neighbours and to the onset of order (consensus). At lower noise, below the transition line we observe either polar or bipolar structuring of the system (Supplementary Fig. 2 B). The range of higher α allows only states with global consensus, while at $\alpha < 0.4\pi$ we see the region of prevalence of the bipolar polarized states. In other words, the polarized states are only possible at low noise (high conformity) and small restriction angles (strongly bounded confidence). The change in level of emotional intensity pushes the boundary between the polarized and non-polarized societies outwards (Supplementary Fig. 2 B), thus extending the range of the polarized states, and brings the originally weakly polarized society to a highly polarized one. The most important observation here is that the transition happens just due to the increase of emotional intensity while the other parameters (conformity, confidence) stay constant. Supplementary Figures 2 C,D show phase diagrams for unparameterized systems at different simulation parameters. For a larger system, $s = 240$ constituting of 58081 agents, the area of bipolar ordering is slightly reduced as compared to the standard system size ($s = 120$) used in this study. The shrinking happens because the influence of biased boundary vectors is decreased due to larger system size. If the agents have larger interaction range, $r = 3$ (i.e. each vectors interacts with up to 48 nearest neighbors), the region of bipolar ordering expands to larger restriction angles and noise values because longer correlation range becomes possible and effect of the boundary bias is more pronounced at these conditions. Finally, if all boundary vectors are fixed in one direction (in this case to the East, $\theta = 0$), the area of bipolar polarization shrinks significantly but does not disappear. At these conditions bipolar ordering also displays more sensitivity with respect to noise than to restriction angle.

Twitter Data

The archived Twitter Streaming API Spritzer Sample tweet data was stored in JSON (**J**ava**S**cript **O**object **N**otation) file format, which is the most common open standard data format to transmit data objects in asynchronous browser/server communication. The data was then processed and analyzed in Python using the Python Natural Language Text Processing Toolkit (NLTK) [1, 2]. NTLK is a collection of various classes, interfaces and functions for natural language processing, including important text mining methods. It was developed at the University of Pennsylvania and is widely used in computational linguistics, machine learning, and cognitive science (see in particular `https://nltk.googlecode.com/svn/trunk/doc/api/index.html`).

The collected tweets were filtered, first for Russian and Ukrainian language, identifying respective alphabet characters in the tweet text. Tweets, where Twitter users specified being from Russia or any region/town in Russia were removed from the data. We also filtered for SPAM tweets (around 17% of all the tweets in our data), using the following keywords, based on word count analyses: porn, phone, games, androidgames, minecraft, ipadgames, loosing weight, gold, mtvstars, crossword, barbie, sony, holidays, shop, TV series, volkswagen, sales, starlet, apartments, estate, bmw, mercedes, sex, happysex, viagra, stock, prostitutes, teen, price, diet, buy, credit in English, Ukrainian and Russian language. Tweets containing these words were removed from the data. Furthermore, we filtered the data for political context with an extensive set of keywords to remove irrelevant tweets and therefore unnecessary noise from the data: protest, mobilization, Ukraine, kiborgi, glory to the heroes (one word hashtag in Ukrainian and Russian - geroiamslava), junta, government, duma, kremlin, parliament, rada, premier ministry, president, minister, ministry, political action, demonstration, opposition, power, authorities, democracy, nationalists, communists, liberals, Putin, Poroshenko, political party, politics, politicians, political, policy, revolution, citizen, criticism, critics, agitation, sanctions, censoring, illegal, legal, solidarity, assembly, rally, law, regulation, resistance, civil disobedience, resist, reforms, communism, capitalism, administration, news, society, violation, RNBO, Iaijtheniukh, ukr, ukry, khokhly, okraina, ruina, raguli, poproshenko, papashenko, papasha, bacon to the heroes (a wordplay in Russian with glory to the heroes - geroiamsala), civil war, nazis, Bandera, banderlogi, bandery, banderovtsy, benderovets, visitka Iarosha, titushki, EuroMaidan, U_revolution, peaceful march, radio freedom, inforesist, Aronets, avtomaijdan, Ukraine truth news (one word hashtag), SOS Maidan (one word hashtag), digital Maidan (one word hashtag), Maidan history (one word hashtag), sidemaidan, Maidan, freedom, Ukrainian, NATO for Ukraine (one word hashtag), will, choice, against, anti-Maidan, ukrop, Europe, USA, Kiev, Timoshenko, Cameron, dead, killed, negotiations, military service, Zakharchenko, police, Obama, war, activists, right sector, Azarov, upper, leader, Jatseniuk, Turchinov, Klitchko, MID, Nayyem, briginets, constitution, Avakov, MVD, Yushenko, Kuchma, Kravchuk, Kharkov, Grushevskogo, warriors, soldiers, prisoners, Euro, EU, military, Sloviansk, army, West, East, Mariupol, reconciliations, NATO, ATO, crimealook, conflict, Medvedchuk, Medvedchukov, geo-politics, confrontation, Strasbourg, crisis, Lutsenko, Tusk, peace, world, Vladimirova, Vladimir, relations, unity, national, help, oligarchs, glory to Ukraine (one word hashtag), hundred, over, own will, self-determination, power, annexion, annexing, separatism, separatist, rebels, freedom fighters, freedom fight, moskali (meaning Moscow sympathizers), [Russian] official, Putin khujlo (offensive word, one word hashtag), luganda, lungandon, daunbas, zrada, occupants, MGB, Zakhar, Russian peace (one word hashtag), Russian world (one word hashtag), Putin is murder (one word hashtag), Putler, Putin help (one word hashtag), putinism, insurgents, insurgency, small/so what Russia, Crimea is ours (one word hashtag), At least Crimea is our (one word hashtag), punitive, ptnpnkh, ptn, pnkh, vata, vatnik, glory to Russia (one word hashtag), Russia, provocation, Novorossiia, DNR, LNR, berkut, kiberberkut, glory, stopcrimeantatarsgenocide, russiainvadeukraine, russiaviolatedceasefire, stoprussianaggression, weapon, Russian, Medvedev, Moscow, legislation, Yanukovich, truth, Donetsk, Luhansk, Russian march (one word hashtag), Russians, Ukrainians, Non-russians, Navalny, monument, Lenin, western, eastern, geek, Lavrov, grad, humanitarian, anti-Russian, SSSR, Donbass, Bafana, elections, legislator, fire, Mirakova, ukraintsami, radio, Putin supporters (one word hashtag), Ukraine_France, Zakharov, Mironov in Ukrainian and Russian language. Tweets that contained at least one of these keywords were kept in the filtered data, otherwise removed.

Any analysis of Twitter data faces a number of well-known difficulties [3]. Some of them, e.g. the SPAM tweet problems, we have addressed already above. One potential problem is that the sample only includes public tweets from public Twitter accounts. This does not pose a problem in the context of our study though, since we are interested in the use of Twitter as an instrument of communication in the public sphere. Moreover, Twitter data is not representative, which again is rather unproblematic for our study because all political groups and their supporters are represented on Twittersphere, so the public debate on Twitter does overall mirror the general public debate and public opinions [4, 5]. Another potential issue is that the sample is based solely on the 1% of all public tweets, which for instance makes it difficult to use the data as panel data (though we've done this to probe opinion changes within individuals, see Supplementary Fig. 3, the number of observations is however drastically reduced). However, other Twitter data samples offered by Twitter (e.g. Gardenhose with 10% of all pubic tweets) have to be purchased, which makes them often unaffordable for research purposes.

One aspect of Twitter data "richness" is that it is supposedly geo-referenced. However, as already mentioned in the main manuscript quite a large proportion of Twitter users do in fact not provide any geographical information. Frequently, even if "geo_enabled" was activated by the Twitter user (thus the value was set to "true"), the actual "geo" or "place" "coordinates" value would be "null" because the device that was used to post the tweet had no geo-referencing (e.g. GPS) activated. The data that the user provides through the profile "location" tag is more often available, however, here we have to rely on the accuracy and honesty of the Twitter users. Moreover, if the information provided through "location" was imprecise, e.g. just "Ukraine", we could not use this information for plotting. Did we however have serious and specific information from the user, e.g. "Kiev", then we used that information to generate a longitude and a latitude coordinate value that we could then plot on a generated Ukraine map in Fig. 3C,D in the main manuscript. We deliberately ignored another potential geographical information, the "time_zone", because it offers only imprecise geographical information. Overall, we could extract form the Twitter data of the last

September week 2014 493 Twitter users for whom we had sufficiently precise geographical data. This data along with the calculated West and East scores were used to produce the Figures 3C and D in the main manuscript.

Twitter Data Analysis

In order to understand the empirical polarization dynamic in Ukraine and furthermore in order to make the data usable for model calibration, it was necessary to identify the political opinion of the Twitter users in our data. First, we identified the Twitter users in our Twitter data based on the value of their "screen_name". We then compiled two lists of keywords, extracted from the word count analysis of the most common words that contain words associated with either the pro-East political camp or the pro-West political camp: annexion: -2 , annexing: -2, separatism: -2, moskali (meaning Moscow sympathizers): -2, [Russian] official: -2, Putin khuijlo (one word hashtag): -3, Luganda: -3, Lugandon: -3, Daunbas: -3, zrada: -2, insurgents: -2, occupants: -2, MGB: 1, Zakhar: 1, Russian peace (one word hashtag): -2, Putin is murder (one word hashtag): -3, Putler: -3, Putin help (one word hashtag): 2, putinism: -1, freedom fighters (one word): 2, freedom fight (one word): 2, small/so what Russia: 2, Crimea is ours (one word hashtag): 3, At least Crimea is ours (one word hashtag): 3, punitive: 3, ptnpnkh: -3, ptn: -1, pnkh: -3, vata: -3, vatnik: -3, glory to Russia: 3, Russia: 0, Russian: 0, Russians: 0, provocation: 0, separatist: -3, separatism: -3, Novorossiia: 2, DNR: 1, LNR: 1, sanctions: 1, berkut: 1, kiberberkut: 1, stopcrimeantatarsgenocide: -3, russiainvadedukraine: -3, russiaviolatedceasefire: -3, anti-Russian sanctions (one word hashtag): 2, stoprussianaggression: -3, peace: 0, Putin: 0, weapon: 0, Medvedev: 0, Crimea: 0, Moscow: 0, Yanukovich: 0, truth: 0, Donetsk: 0, from Lugansk (one word): 0, Russian march (one word hashtag): 0, Non-Russians: 0, dead/killed: 0, negotiations: 0, politce: 0, rebels: 0, Lugansk: 0, war: 0, duma: 0, Mockva: -1, government: 0, leader: 0, monument: 1, Lenin: 1, warriors: 0, prisonerns: 0, military: 0, East: 0, army: 0, relations: 0, soldier: 0, reconciliations: 0, geek: -2, Lavrov: 0, grad: -1, humanitarian: 1, anti-Russian: 2, SSSR: 1, Donbass: 0, Bafana: 0, elections: 1, referendum: 0, confrontation: 0, fire: 0, crisis: 0, conflict: 0, Putin supporters (one word hashtag): -1, Mironov: 0 were the keywords for the East political camp and mobilization: 1, kiborgi: 2, unity: 1, glory to the heroes (one word hashtag): 3, RNBO: 1, Iaijtheniukh: -3, junta: -2, ukr: -3, ukry: -3, khokhly: -1, okraina: -1, Ruina: -3, raguli: -3, poproshenko: -2, Papashenko: -2, papasha: -3, bacon to the heroes (a wordplay in Russian with glory to the heroes - geroiamsala): -3, civil war (one word hashtag):- 2, visit Kaiarosha (one word hashtag): 2, nazis: -3, Bandera: -3, banderlogi: -3, bandery: -3, banderovtsy: -3, titushki: 2, Ukraine: 0, Euro Maidan (one word hashtag): 1, U_REVOLUTION: 2, peaceful march (one word hashtag): 1, radio freedom (one word hashtag): 1, inforesist: 1, Aronets: 1, avtomaijdan: 1, Ukrainian truth news (one word hashtag): 1, SOS Maidan (one word hashtag): 1, digital Maidan (one word hashtag): 1, Maidan history (one word hashtag): 1, sitemaidan:1, Maidan: 0, freedom:1, government: 1, Ukrainian: 0, Urkrainians: 0, NATO for Ukraine (one word hashtag): 2, will: 2, will: self-determination, against: 2, revolution: 2, anti-Maidan:- 2, ukrop: -3, Poroshenko: 0, Europe: 0, USA: 0, Kiev: 0, weapon: 0, Timoshenko: 0, truth: 0, Cameron: 0, democracy: 0, dead/killed: 0, negotiations: 0, military service: 0, Zakharchenko: 0, opposition: 0, Obama: 0, war: 0, parliament: 0, activists: 0, political action (one word): 0, protest: 0, sector: 0, Azarov: 0, upper: 0, leader: 0, rada: 0, Yatseniuk: 0, Turchinov: 0, Klichko: 0, MID: 0, Nayyem: 0, Briginets: 0, constitution: 0, president: 0, Avakov: 0, MVD: 0, Yushchenko: 0, Kuchma: 0, Kravchuk: 0, Kharkov: 0, Grushevskogo: 0, warriors: 0, Euro: 0, EU: 0, military: 0, Slaviansk: 0, West: 0, western: 0, army: 0, soldier: 0, Mariupol: 0, reconciliations: 0, NATO: 0, ATO: 1, conflict: 0, Medvedchuk: 0, Medvedchukvv: 0, Strasbourg: 0, crisis: 0, Lutsenko: 0 ,Tusk: 0, peace: 0, relations: 0, national: 1, help: 1, glory to Ukraine (one word hashtag): 2, hundred: 2, over: 2, power: 2, BENDERovets: 3, civil war: -2, krimealook: 1, Ukraine_France: 1 were the keywords for the West political camp. These keywords were scored between -3 and 3, with negative scores indicating negative attitudes towards the respective political camp, positive values positive attitudes and a zero a neutral opinion. Some keywords were unambiguously associated with a political affiliation. For instance, the word "Putler" (scored -3), a composition of Putin and Hitler, is clearly a negatively annotated word referencing the East political camp. Similarly, the word "banderovtsy" (scored -3) linking the West political camp to Stepan Bandera, leader of the Ukrainian nationalist and independence movement during the Second World War, who cooperated with Nazi Germany, shows clearly a strong disapproval of the West political camp. On the other hand the hashtag #slavarossii (translated: Glory to Russia, scored +3), shows a clear support for the East political camp, while the hashtag #natoforukraine (scored +3) expresses an unequivocal pro-Western political affiliation. A word like "Putin" (East political camp) or "Poroshenko" (West political camp) however would be assigned a "0" because depending on the remaining content of the tweet the names could have been associated with positive or negative attitudes. And for that reason an additional sentiment analysis of the tweets was necessary.

We used the sentiment analysis SentiStrength (`http://sentistrength.wlv.ac.uk`) approach to determine a sentiment score for each tweet. SentiStrength is a free Java-based automatic sentiment analysis tool, widely used in research, which is also available for the Russian language. We created a comprehensive dictionary for Ukrainian sentiment words (to be shared upon request) based on the SentiStrength sentiment scoring system and we reviewed the sentiment word dictionary that SentiStrength is using for the Russian language and complemented it with other

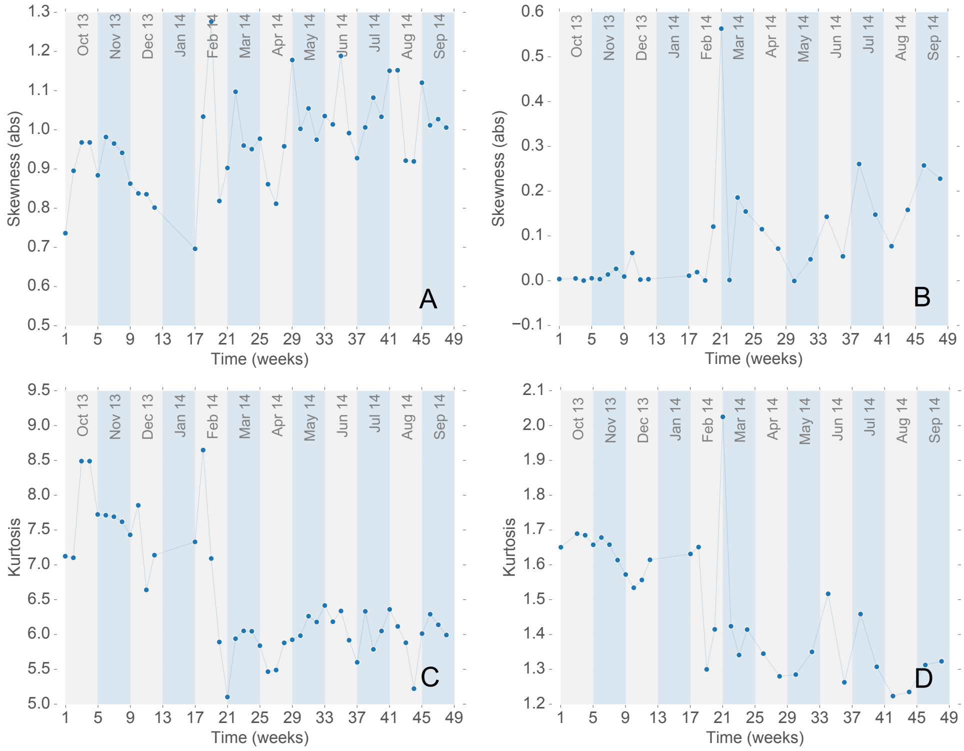

Supplementary Figure 4: (A) Time series plot of absolute skewness for opinion distributions in Twitter data. (B) Time series plot of absolute skewness for opinion distributions in simulations. (C) Kurtosis of the distribution of opinions in Twitter data. (D) Kurtosis of the distribution of opinions in simulations. Simulation parameters in plots B and D are $\alpha = 0.15$ and $\eta = 0.12$.

sentiment words (among others offensive words that were missing in SentiStrength) to make it equivalent to the Ukrainian dictionary. The sentiment scores in SentiStrength and thus in our two sentiment words dictionaries range from -5 to +5 with 0 signifying a neutral word, negative values a negative sentiment and positive values a positive sentiment. The higher the absolute value the stronger the sentiment. For each tweet we calculated a sentiment score average based on the identified sentiment words in the tweet. We calculated a separate sentiment score for the East political camp related keywords and one for the West political camp related keywords. The emotional intensity score is averaging these two sentiment scores.

In the case of unequivocal keyword tweets, the tweet score would derive from these keywords. For instance, if a keyword contained the words "Putler" (scored -3) and the hashtag "stoprussianaggression" (scored -3), the tweet score would be the average of these two scores, thus -3. Whenever a tweet was scored zero because of neutral keywords, we added to the score the respective sentiment score. Thus if an East political camp related tweet had a zero score (e.g. "Putin"), and the East political camp related sentiment score was -2 (for instance resulting from the sentiment word "zachvatil" (translated: grabbed)), then the tweet would get an East political affiliation score of -2. This is based on the assumption that users would express positive sentiments about terms associated with their own camp and/or negative sentiments towards terms associated with the other camp. Since most users would have posted several tweets, users were assigned a set of tweet scores, depending on the number of tweets and from these scores overall average scores, one East score, one West score, were calculated for each user, representing their political affiliation

Automatic classification and sentiment analysis have certainly their limitations, e.g. automatic sentiment analysis often fail to spot irony and classifications do not account for instance for complex inner-fraction dynamics, that is political fractions within political fractions. However, manual classification is becoming increasingly impossible with the growing amount of data and/or limited capacities and resources and thus automatic classification is increasingly applied. It is however important to work on further developing and elaborating these tools and to supervise, critically reflect and where required correct the process of automatic analysis and its outcomes.

Additional Results

We present here some additional results that support our main conclusions in the manuscript. Supplementary Figure 4 shows the skewness and kurtosis of opinions in the Twitter data and computer simulation. These plots show again the discontinuity of political opinions in the Ukrainian Twittersphere, with jumps in February 2014.

[1] S. Bird, E. Klein, and E. Loper, *Natural Language Processing with Python* (Sebastopol: O'Reilly, 2009).
[2] J. Perkins, *Python Text Processing with NLTK 2.0 Cookbook* (Birmingham: PACKT, 2010).
[3] D. Ruths and J. Pfeffer, Science **346**, 1063 (2014).
[4] D. Stern, *The Twitter war: Social media's role in Ukraine unrest*, `http://news.nationalgeographic.com/news/2014/05/140510-ukraine-odessa-russia-kiev-twitter-world/` (2014), accessed on: 30 May 2017.
[5] A. Ronzhyn, in *Proceedings of the European Conference on Social Media: ECSM 2014* (2014).
